# The crucial role of bioimage analysts in scientific research and publication

**DOI:** 10.1242/jcs.262322

**Published:** 2024-10-30

**Authors:** Beth A. Cimini, Peter Bankhead, Rocco D'Antuono, Elnaz Fazeli, Julia Fernandez-Rodriguez, Caterina Fuster-Barceló, Robert Haase, Helena Klara Jambor, Martin L. Jones, Florian Jug, Anna H. Klemm, Anna Kreshuk, Stefania Marcotti, Gabriel G. Martins, Sara McArdle, Kota Miura, Arrate Muñoz-Barrutia, Laura C. Murphy, Michael S. Nelson, Simon F. Nørrelykke, Perrine Paul-Gilloteaux, Thomas Pengo, Joanna W. Pylvänäinen, Lior Pytowski, Arianna Ravera, Annika Reinke, Yousr Rekik, Caterina Strambio-De-Castillia, Daniel Thédié, Virginie Uhlmann, Oliver Umney, Laura Wiggins, Kevin W. Eliceiri

**Affiliations:** ^1^ Imaging Platform, Broad Institute of MIT and Harvard, Cambridge, MA 02142, USA; ^2^ Edinburgh Pathology, Centre for Genomic & Experimental Medicine and CRUK Scotland Centre, Institute of Genetics and Cancer, The University of Edinburgh, Edinburgh EH4 2XU, UK; ^3^ Crick Advanced Light Microscopy STP, The Francis Crick Institute, London NW1 1AT, UK; ^4^ Department of Biomedical Engineering, School of Biological Sciences, University of Reading, Reading RG6 6AY, UK; ^5^ Biomedicum Imaging Unit, Faculty of Medicine and HiLIFE, University of Helsinki, FI-00014 Helsinki, Finland; ^6^ Centre for Cellular Imaging, Sahlgrenska Academy, University of Gothenburg, SE-405 30 Gothenburg, Sweden; ^7^ Bioengineering Department, Universidad Carlos III de Madrid, 28911 Madrid, Spain; ^8^ Center for Scalable Data Analytics and Artificial Intelligence (ScaDS.AI) Dresden/Leipzig, Universität Leipzig, 04105 Leipzig, Germany; ^9^ DAViS, University of Applied Sciences of the Grisons, 7000 Chur, Switzerland; ^10^ Electron Microscopy STP, The Francis Crick Institute, London NW1 1AT, UK; ^11^ Fondazione Human Technopole, 20157 Milan, Italy; ^12^ Science for Life Laboratory BioImage Informatics Facility and Department of Information Technology, Uppsala University, SE-75105 Uppsala, Sweden; ^13^ Cell Biology and Biophysics, European Molecular Biology Laboratory, 69115 Heidelberg, Germany; ^14^ Randall Centre for Cell and Molecular Biophysics and Research Management & Innovation Directorate, King's College London, London SE1 1UL, UK; ^15^ GIMM - Gulbenkian Institute for Molecular Medicine, R. Quinta Grande 6, 2780-156 Oeiras, Portugal; ^16^ La Jolla Institute for Immunology, Microscopy Core Facility, San Diego, CA 92037, USA; ^17^ Bioimage Analysis & Research, BIO-Plaza 1062, Nishi-Furumatsu 2-26-22 Kita-ku, Okayama, 700-0927, Japan; ^18^ Institute of Genetics and Cancer, The University of Edinburgh, Edinburgh EH4 2XU, UK; ^19^ University of Wisconsin-Madison, Biomedical Engineering, Madison, WI 53706, USA; ^20^ Image Analysis Collaboratory, Harvard Medical School, Boston, MA 02115, USA; ^21^ Nantes Université, CHU Nantes, CNRS, Inserm, BioCore, F-44000 Nantes, France; ^22^ Minnesota Supercomputing Institute, University of Minnesota Twin Cities, Minneapolis, MN 55005, USA; ^23^ Åbo Akademi University, Faculty of Science and Engineering, Biosciences, 20520 Turku, Finland; ^24^ Pixel Biology Ltd, 9 South Park Court, East Avenue, Oxford OX4 1YZ, UK; ^25^ Scientific Computing and Research Support Unit, University of Lausanne, 1005 Lausanne, Switzerland; ^26^ Division of Intelligent Medical Systems and Helmholtz Imaging, German Cancer Research Center (DKFZ), 69120 Heidelberg, Germany; ^27^ Université Grenoble Alpes, CNRS, CEA, IRIG, Laboratoire de chimie et de biologie des métaux, F-38000 Grenoble, France; ^28^ Université Grenoble Alpes, CEA, IRIG, Laboratoire Modélisation et Exploration des Matériaux, F-38000 Grenoble, France; ^29^ Program in Molecular Medicine, University of Massachusetts Chan Medical School, Worcester, MA 01605, USA; ^30^ Institute of Cell Biology, The University of Edinburgh, Edinburgh EH9 3FF, UK; ^31^ BioVisionCenter, University of Zurich, 8057 Zurich, Switzerland; ^32^ School of Computing, University of Leeds, Leeds LS2 9JT, UK; ^33^ University of Sheffield, Department of Materials Science and Engineering, Sheffield S10 2TN, UK

**Keywords:** Bioimage analysis, Bioimage analysts, Bioimaging, Training

## Abstract

Bioimage analysis (BIA), a crucial discipline in biological research, overcomes the limitations of subjective analysis in microscopy through the creation and application of quantitative and reproducible methods. The establishment of dedicated BIA support within academic institutions is vital to improving research quality and efficiency and can significantly advance scientific discovery. However, a lack of training resources, limited career paths and insufficient recognition of the contributions made by bioimage analysts prevent the full realization of this potential. This Perspective – the result of the recent The Company of Biologists Workshop ‘Effectively Communicating Bioimage Analysis’, which aimed to summarize the global BIA landscape, categorize obstacles and offer possible solutions – proposes strategies to bring about a cultural shift towards recognizing the value of BIA by standardizing tools, improving training and encouraging formal credit for contributions. We also advocate for increased funding, standardized practices and enhanced collaboration, and we conclude with a call to action for all stakeholders to join efforts in advancing BIA.

## Teaser

**Summary:** We examine the importance of bioimage analysis, obstacles to wider adoption of these important methods and ways to ensure that bioimage analysts receive the career support they need.

## Introduction

### Bioimage analysis as an emerging discipline

Computational analysis of image data generated with techniques such as light and electron microscopy, pre-clinical imaging, and clinical imaging is a comparatively recent development in the centuries-long history of light microscopy for biological discovery. To interpret biological images, scientists have long relied on the human visual system, which is not suited for reproducible quantification (Jambor, 2023). The subjective nature of visual analysis has famously been demonstrated in neuroscience, where Golgi and Cajal used the same method to reach widely different conclusions regarding neuroanatomy, and is perhaps best seen in pathology, where despite a long history and comprehensive training of researchers in discerning phenotypes from images, the interpretation of many phenotypes results in poor diagnostic agreement between individual observers (Elmore et al., 2017; Hamilton et al., 2015; Polley et al., 2013; Varga et al., 2012). Such disagreement, alongside the limitations of human perception in detecting subtle phenotypes (Gibson et al., 2015), underlies the serious need for quantitative, reproducible methods in bioimage analysis (BIA) that align with the FAIR (findable, accessible, interoperable and reusable) principles for scientific data management and stewardship (Barker et al., 2022; Kemmer et al., 2023; Wilkinson et al., 2016).

Further complicating this landscape is the sheer number of advanced microscopy techniques. Microscopes are both ubiquitous and extraordinarily heterogeneous. This combination means that, although microscopy has a low barrier to entry, standardization of methods poses a significant challenge, leading to the emergence of an entire career track for microscopy experts: the imaging scientist (Wright et al., 2024). The heterogeneity of microscopes and microscopy applications is naturally reflected in the imaging data itself; therefore, extensive time and expertise are required to design and implement robust BIA methods. The 2010s saw the rise of network organizations, such as the Network of European BioImage Analysts (NEUBIAS), aiming to connect experts in the creation and application of BIA tools (Martins et al., 2021). Although artificial intelligence (AI) and machine learning (ML) will continue to dramatically improve the throughput of BIA and create newer, easier-to-use analytical options, the human expertise of imaging scientists and bioimage analysts is still needed to provide the detailed understanding necessary to both generate viable training datasets and implement these techniques correctly.

**Fig. 1.**
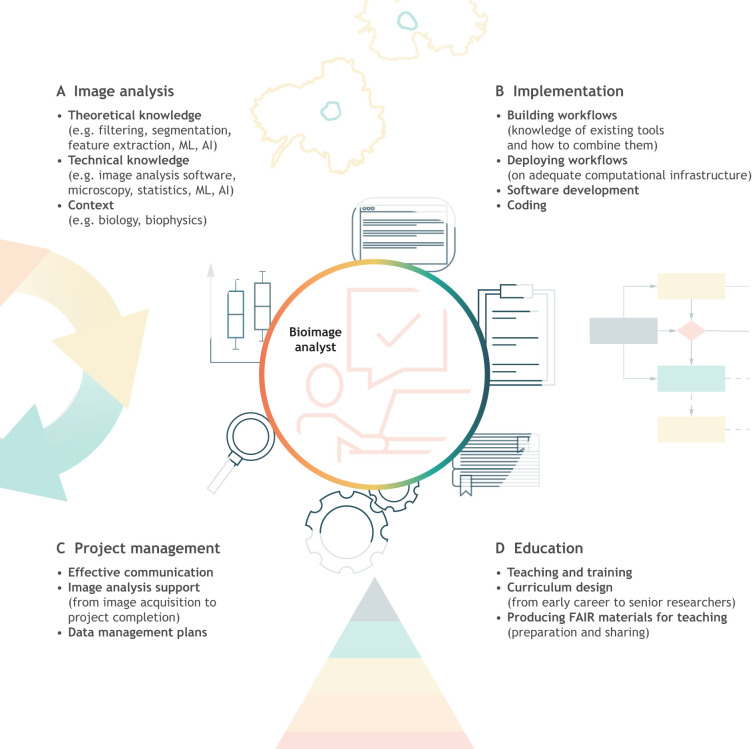
**Overview of the key skills and capabilities of a BIA specialist.** Four categories of skills and capabilities have been identified: (A) image analysis, including theoretical and technical knowledge as well as a good understanding of the context of scientific questions; (B) implementation, covering building and deploying computational workflows, developing pipelines, and coding; (C) project management, including communicating effectively, providing support and devising data management plans; (D) education, focusing on teaching and training, designing curricula, and producing materials.

We stand at a critical juncture where the establishment of dedicated BIA support within academic and research institutions – in the form of expert groups, facilities and dedicated staff in individual laboratories – presents a huge opportunity to enhance the quality and efficacy of research output. Since the conclusions of an experiment ultimately rest on its design, the inclusion of BIA experts during the experimental design phase can make the difference between a successful or an unsuccessful outcome – as Ronald Fisher said, “To consult the statistician after an experiment is finished is often merely to ask him to conduct a post-mortem examination. He can perhaps say what the experiment died of.” Direct contributions from BIA experts lead to better data utilization, more principled and conclusive results, stronger publications, and a highly productive environment that is conducive to fewer revision cycles and improved project setups, thus directly impacting scientific endeavors (Fig. 1) (Jambor et al., 2021; Jonkman et al., 2020; Lee et al., 2024; Senft et al., 2023; Soltwedel and Haase, 2024). They can also help guard against research misconduct (Bik et al., 2016; Pulverer, 2015; Rossner and Yamada, 2004), the majority of which is thought to be inadvertent and due to insufficient understanding of best practices (Pulverer, 2015). Bioimage analysts thus play a pivotal role in elevating the scientific reputation of an institution, attracting superior talents and nurturing a vibrant community that pushes scientific frontiers. Nevertheless, we estimate that most biological researchers do not actively consult BIA experts. Although to our knowledge no study has determined why this is, in our experience, low computational comfort (resulting in discomfort when approaching computational experts), lack of awareness about manual analysis biases (Lee et al., 2024), lack of awareness of available methods, lack of knowledge about how to find BIA experts and lack of resources to hire BIA experts all contribute to the relatively low rate of collaboration.

The BIA field is technologically poised for a paradigm shift. As ML approaches increasingly allow automation of routine tasks such as segmentation, future BIA specialists will be free to focus on experimental design and advanced method development and application. Culturally, however, this evolution will require a shift towards a more inclusive, collaborative approach, where bioimage analysts participate in the experimental design process from the outset. A concerted effort to foster a culture that recognizes the intrinsic value of quantitative BIA and the indispensable role of analysts in advancing scientific discovery will allow many positive returns, including deeper scientific insights and more efficient research methodologies. In this Perspective, we describe the results of a recent meeting of bioimage analysts at The Company of Biologists ‘Effectively Communicating Bioimage Analysis’ Workshop, held in February 2024. This Workshop hosted BIA practitioners, tool developers and method developers, and served as a platform for discussions in which we categorized the current obstacles and possible solutions to the creation of such a new BIA culture, as detailed below.

## Educating biological researchers about BIA

**Fig. 2.**
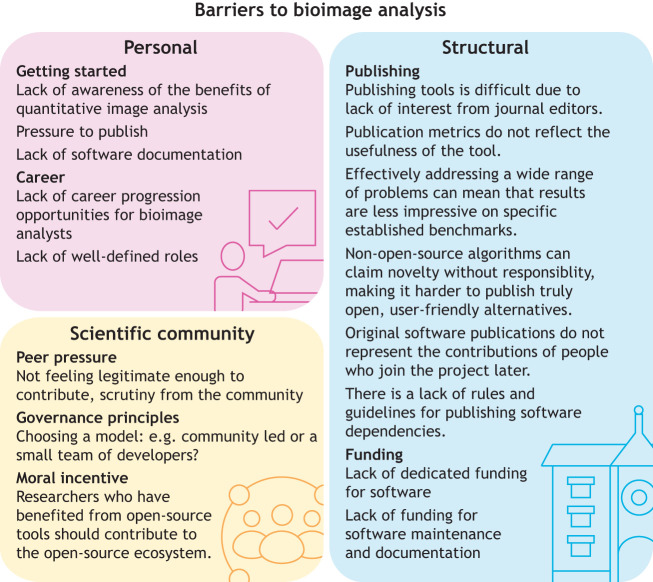
**Major barriers to effective uptake of BIA.** We have identified three categories of barriers: personal barriers, including the difficulties related to getting started in BIA and to finding suitable career options; structural barriers, including barriers in publishing and obtaining funding; and barriers related to the culture of the scientific community, such as peer pressure, a lack of incentives and a lack of clear governance principles.

Advancing BIA as a discipline will require the broader biology community to become more engaged in using BIA in their work. In addition to the cultural barriers described above, several other factors currently limit the ability of many researchers to perform high-quality and FAIR BIA in biology. Biologists who lack education or support in BIA might not be sure which tools to invest their limited analysis time into, and a lack of documentation and proper training materials available for BIA tools might discourage them from delving deeper. Researchers engaged in BIA also often encounter technological obstacles, such as insufficient computing power, storage space or funding for commercial licenses (Fig. 2). The help of a BIA expert and access to more powerful hardware and/or software is therefore often critical to a researcher's success, but these barriers also highlight the need to provide biologists with expanded training opportunities in programming, empowering them with necessary skills.

**Fig. 3.**
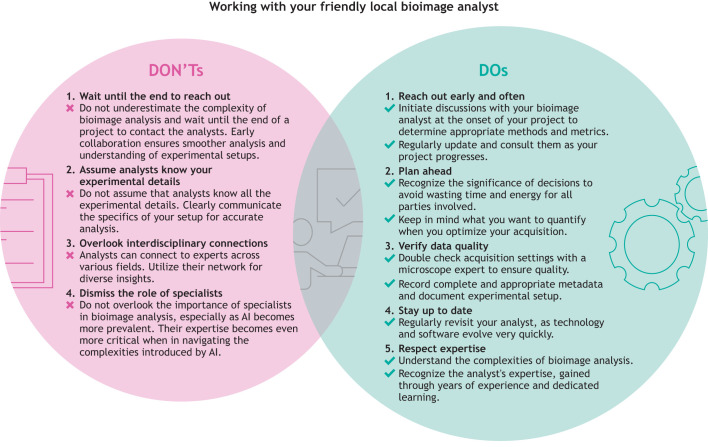
**A concise etiquette guide for interacting with BIA specialists.** We recommend contacting your local bioimage analyst from the start of the project and providing them with regular updates instead of waiting for a complete acquired dataset before reaching out. Moreover, during the data acquisition phase, we suggest that experimentalists prepare a detailed plan for better time management and to aid effective communication with BIA specialists. Additionally, we ask for the bioimage analyst's expertise to be respected and for their scientific contributions to be recognized.

Effective BIA requires a comprehensive understanding of several diverse fields, including image processing, complex workflow creation (Cimini, 2024; Miura and Nørrelykke, 2021) and, increasingly, familiarity with data management, IT infrastructure and deep learning (Fig. 1). At each step, algorithm selection and/or defining large, complex parameter sets may be required. This technical understanding must be paired with thorough understanding of experimental design, including which artifacts are likely under various sample preparations and/or imaging conditions and how best to ameliorate them (Culley et al., 2024; Senft et al., 2023). Statistical considerations, such as measurement errors associated with image analysis, what data should be used to train or validate an algorithm, selection of appropriate metrics, whether and how to aggregate data (including how variability and uncertainty should be assessed), and how to report the results so that they are reproducible and interpretable, are also necessary. Only with all of this knowledge can bioimage data be accurately analyzed, underlining the importance of consultation throughout the experimental design process (Fig. 3).

Bioimage analysts are typically also on the front line of creating FAIR training materials that facilitate learning but also promote the sharing and standardization of best practices within the community (Haase et al., 2024a preprint; Imreh et al., 2024). The creation of standardized, quality-controlled, modularized educational materials would improve the ability of all bioimage analysts to effectively train diverse audiences, from novices to advanced practitioners, with tailored curricula both in formal educational settings and in more informal workshops. Community efforts (including funding acquisition) should also be undertaken to develop training schools and curricula in which one can ‘train the trainers’. Importantly, BIA training materials must be made accessible to a diverse audience and ideally be understandable for researchers with various backgrounds. This will require adjustments for individuals with disabilities, translations into multiple languages to reach a global audience, and inclusion of a variety of formats ranging from informal blog posts and instructional videos to full academic courses. In addition, a collection of case studies that illustrate the practical application of BIA skills in various research contexts could be compiled to serve as a valuable resource for both learners and trainers.

To facilitate this, existing material should be reviewed (for example, surveys and meta analyses of Jamali et al., 2022; Miura, 2021; Schmidt et al., 2022; Sivagurunathan et al., 2023; Waithe, 2021) and cataloged, allowing greater focus to be placed on missing elements important for FAIR training. These could include, but not be limited to: application of ML techniques in BIA for more efficient and accurate image processing and analysis; ethical considerations, data privacy and the responsible use of bioimage data, especially in contexts where sensitive or personal information may be involved; how to perform correct validation and accurately report and interpret results; proper use of statistics, including how to report *P*-values and prevent ‘*P*-hacking’; training on the use of IT infrastructure, such as high-performance computing and cloud computing; and research data management and reporting guidelines (Sarkans et al., 2021; Schmidt et al., 2022, 2024), with BIA-specific data management plans containing sections about who is responsible for processing the data, from where they get their resources (including human resources, storage resources and computing resources) and how to ‘FAIR-ify’ the project (such as by sharing data and code sustainably).

In tandem, the BIA community can lead a concerted effort to develop better standards and harmonized approaches with the goal of simplifying the learning process, making it easier to train future analysts and researchers. This will require engagement from those across the BIA community, including researchers, developers, funders and policy makers. Fostering interoperability through the use of common file formats (Hiner et al., 2016; Moore et al., 2021), the creation and adoption of metadata formats that include aspects of experimental design, and promoting best practices in software engineering for greater modularity and documentation (Afiaz et al., 2024; Sharma et al., 2024; Sivagurunathan et al., 2023; Wiesmann et al., 2015) will ensure that image data can be used and re-used with many tools. The establishment of standardized datasets and metrics for tool evaluation (Maier-Hein et al., 2024; Rubens et al., 2020) as well as the benchmarking initiatives these enable – such as setting up algorithm challenges for solutions to BIA problems like segmentation and object tracking (Caicedo et al., 2019; Haase et al., 2024b preprint; Ma et al., 2024; Maška et al., 2023; Ulman et al., 2017) – can enhance visibility and comparability among tools and reduce duplicated efforts. By adopting these strategies, the BIA community can foster a more integrated, efficient and collaborative research environment.

## Enabling sustainable career paths in BIA

The broad technical knowledge required to successfully become a BIA expert typically takes many years to develop, especially in the absence of formalized training pathways. As such, bioimage analysts are sometimes employed in academic core facilities, like other technical specialists such as bioinformaticians and imaging scientists. Because they are often exposed to a range of biological problems, broad subsets of the BIA tool ecosystem and user difficulties with said tools, such ‘BIA application experts’ can highlight the unmet needs of the biological community when communicating with (or even serving as) research software engineers (Deschamps et al., 2023). To succeed in such roles, bioimage analysts must possess not only the technical skills described above but also the ability to understand and communicate analysis techniques in order to be able to advise and train researchers on the requirements and resources for data sharing (Fig. 1). As detailed above, the benefits to institutions employing such individuals are vast, as they can raise the quality of research for whole departments through consultations on analyses and statistical presentation of image data, as well as facilitate more efficient use of computational resources through consultation on data management and cluster utilization, reducing institutional costs.

In our collective experience, few people currently have all the skills needed to succeed in this career, and those who do might find the academic core facilities career path to be challenging and unstable. Although in some regions, such as parts of Europe, it is common to find regional or national funding for core facilities staff (O'Toole and Marrison, 2024; Pfander et al., 2022), in other regions, such as the United States, this type of funding is difficult if not impossible to acquire, leaving the funding of core facility staff up to individual institutions. In a recent Global BioImage Analysts' Society (GloBIAS) survey of the field (GloBIAS Survey Working Group, personal communication), fewer than 20% of bioimage analysts working in core facilities reported that their facility is more than 75% internally funded, suggesting that most institutions do not have sufficient funding dedicated to covering salary and expenses for bioimage analysts. Most analysts rely on a combination of collaborators’ grants and microscope usage fees to make up the remainder of the budget, with a smaller fraction of money coming from their own grants, external consulting or fees from analysis projects. Only one of 166 respondents reported being able to fund more than 75% of their position through image analysis fees alone, which likely reflects similar cost recovery challenges in other kinds of computational core facilities due to higher salaries of computational experts and lower willingness of researchers to pay for computational work (Dragon et al., 2020). Without reliable institutional or agency funding, academic core facility bioimage analysts typically have little job security, low pay and the same difficulties in career progression found in other core facility career paths (Adami et al., 2021; Dragon et al., 2020; Lippens et al., 2022; Rahmoon et al., 2024; Soltwedel and Haase, 2024; Tranfield and Lippens, 2024; Waithe, 2021; Wright et al., 2024).

Unfortunately, the historical reliance on qualitative assessment in biology research has led to relative underdevelopment and underappreciation of the difficulty of BIA, combined with other cultural biases against facility technical specialists (Knudtson et al., 2019; Kos-Braun et al., 2020) (Fig. 3). Researchers often approach bioimage analysts with predetermined mindsets about what an analysis should look like and what the results ought to be, rather than engaging in a collaborative dialogue to explore the full spectrum of analytical tools available. This disconnect not only suppresses innovation in novel methods and approaches but also prevents the optimal application of BIA in addressing complex scientific questions. In many scientific environments, bioimage analysts find their contributions condensed to brief mentions in the methods section or relegated to supplementary materials despite their pivotal role in shaping the research outcome from the outset. This oversight not only diminishes the value of their expertise but also risks diminishing the integrity and reproducibility of scientific findings. The combination of low pay and under-acknowledgement can lead to burnout in such analysts, who often find themselves both more respected and better compensated in industry roles. This leads to a ‘brain drain’ in which highly talented experts who serve as a critical resource for both researchers and BIA tool creators are often lost from the academic community.

It will therefore be vital to establish a stable career path for BIA specialists. BIA experts (alongside their imaging scientist colleagues) represent a crucial, stable source of knowledge and expertise within their local community. Increasing the number of BIA experts, both by expanding existing training efforts (Cimini et al., 2024; Martins et al., 2021) and by promoting better career paths and acknowledgement structures for analysts, will be essential to generating the expertise needed to push imaging science forward. Other types of core facilities can serve as a template for ways to promote career advancement as well as adoption of training standards and programs (Adami et al., 2021; Waters, 2020; Wright et al., 2024). Internal funding for such facilities typically requires convincing stakeholders of the value of such a facility; therefore, quantifying the benefits and contributions of BIA in measurable terms for funders poses a challenge, as each institution or granting agency will differently value key performance indicators such as impact factor, publication timelines or production of open data. Within universities, academic stakeholders must recognize the value of on-site BIA experts who can train users and propose tailored solutions over purchasing proprietary software with limited scope and user training resources. Here, we provide a draft template (Table S1) to help bioimage analysts (or those wishing to employ them) translate their many roles into monetary value. Although this template must be personalized for every situation, it can be used in discussions on key performance indicators with decision makers to help explain the value of a dedicated BIA facility (Soltwedel and Haase, 2024). While our template covers many possible activities, the number and priority of aspects that any given facility can cover should be tailored to the staffing size, as the requirement to cover too broad an area of expertise is a known issue affecting staff retention and well-being in other bioinformatics facilities (Dragon et al., 2020).

The creation of dedicated BIA core facilities and/or the embedding of bioimage analysts in bioimaging core facilities can help institutions centralize and share costs for these services, which are increasingly integral for the functioning of departments or even whole universities. In accordance with common funding models used in both imaging core facilities and bioinformatics facilities (Dragon et al., 2020; O'Toole and Marrison, 2024; Soltwedel and Haase, 2024; Tranfield and Lippens, 2024; Waithe, 2021), bioimage analysts can of course generate revenue but also should not be required to recover all of the associated costs through user fees. Costing model choices are absolutely critical. Charging per project can limit access to only higher-funded labs, whereas a centrally funded BIA facility with set hours per group can provide a more equitable service (Soltwedel and Haase, 2024). Since BIA needs are often unexpected, this model also protects groups who find themselves suddenly needing BIA services but who had not previously budgeted for them and might not realize the costs required for computational collaboration. For projects requiring significant research from BIA experts, the intellectual contributions of such experts should be recognized by including them as co-principal investigators on grants. To prevent the unfortunate but not uncommon practice of including computational collaborators on initial grants only to make massive cuts to their budget once a grant is awarded (Way et al., 2021; https://www.timeshighereducation.com/opinion/scientific-collaborators-are-not-disposable), BIA co-principal investigators should be provided with official subcontracts that cannot be reduced without mutual agreement.

The increasing inclusion of datasets, methods and software sections in peer-reviewed papers by journals is a welcome development that allows BIA experts more opportunities to publish their work; however, we emphasize that academic citations should only be viewed as one metric of value, as we will detail below. Alternative mechanisms of recognition – such as the increased use of narrative CVs emphasizing the value of collaboration and support work, and the creation of field recognition structures – will also allow hiring and promotion committees to recognize excellence and community value in BIA. The BIA community could work to create such awards to foster recognition of innovation, collaboration and excellence and to acknowledge the diverse contributions of its members, from enthusiastic students to seasoned principal investigators. Creation of awards that highlight impactful work in BIA across all levels of scientific engagement could be administered by reputable entities within the community, including societies like GloBIAS, funders like the Chan Zuckerberg Initiative, or individual institutes and universities.

## Encouraging a culture shift around the importance of BIA

BIA experts who develop new software tools, much like those who work with researchers on BIA applications, are also undervalued in many academic structures and need better working conditions that will inspire them to develop, maintain and update those tools, including a ‘critical mass’ of local experts to interact with (Way et al., 2021). Unfortunately, the current incentive structure in academic research makes it harder than necessary to build and support the practical BIA tools that the wider community needs (Fig. 2).

One of the main quantified outputs of academic research is peer-reviewed publications (Derrick et al., 2024; Way et al., 2021). The ‘publish or perish’ paradigm encourages researchers to narrowly focus on their own area of expertise rather than target their tools broadly. BIA tool developers are thus expected to continually publish novel methods that demonstrably improve on the state of the art according to some benchmark – even though novelty and benchmark performance are not reliable surrogate measures of real-world usefulness (Maier-Hein et al., 2018). Additionally, although a ‘proof-of-concept’ algorithm that works on a restricted dataset might be publishable, there is often no requirement for authors to provide any code that would enable scrutiny or reproducibility of the method (Sharma et al., 2024). Compounding this, academic research labs are typically primarily staffed by trainees, who are unlikely to continue maintaining software that they generated in previous positions.

Making an algorithm accessible to researchers can also be a double-edged sword: a BIA specialist that takes the considerable time and effort needed to create well-documented, user-friendly, open-source software will end up with fewer publications, may be falsely perceived as less productive and might have their work dismissed as ‘software, not research’. If they attempt to incorporate an algorithm into a larger tool within the BIA ecosystem rather than make a new tool, it might be falsely perceived as a trivial advance even when its functionality is novel. The payoff from such efforts is also unclear: even when software is broadly used by non-computational users, a commensurate level of citation is not guaranteed (Giving software its due, 2019). The amount of overlap and redundancy in these tools not only dilutes resources but can also have a considerable environmental impact due to the computational resources required to retrain deep learning models for slight advancements. Current incentives therefore create a ‘graveyard’ of unmaintained, standalone ‘usable enough’ and ‘good enough’ tools developed for particular projects rather than reusable workflows or plugins for existing BIA tools that would promote FAIR principles. (Deschamps et al., 2023; Moses and Pachter, 2022).

The BIA field would thus strongly benefit from funding dedicated to encouraging software maintenance and documentation, which are essential to users, rather than novelty alone. Some philanthropic funders have introduced such programs (such as Chan Zuckerberg Initiative Imaging Software Fellows), but far more of this type of funding is needed globally to promote the long-term maintenance and support of existing solutions rather than the recurrent cycle of development and abandonment. Such a shift in funding models should be accompanied by community effort to define criteria for quality software and good practices for software development and maintenance. Taken as a whole, these measures could help direct efforts towards a smaller number of BIA tools, with the benefits of greatly improved quality, accessibility and usability.

## Conclusions and outlook

**Fig. 4.**
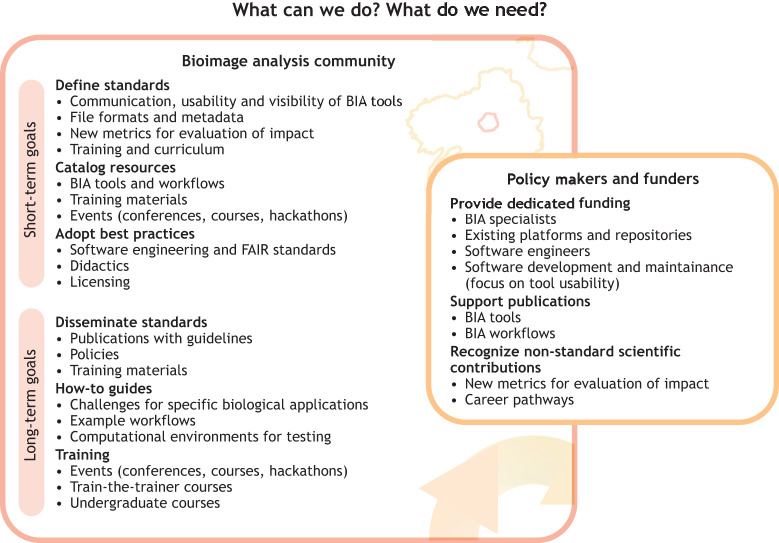
**A vision for** the future of the **BIA community.** We have set short-term and long-term goals for our community to address. In the short term, we would like to direct our efforts into defining standards, cataloging resources and routinely adopting best practices. In the long term, the defined standards should be disseminated through publications, policies and training. These objectives need the support of policy makers and funders for success. A particular focus should be placed on tailored funding opportunities, publication support and recognition of non-standard scientific contributions.

Although significant steps have been taken in creation and adoption of BIA as an expert discipline, many challenges remain. Given the complexity and diversity of biological data, coupled with the rapid evolution of imaging technologies, a concerted effort to use BIA to advance scientific knowledge is necessary. This requires the collaborative engagement of all stakeholders involved in the bioimaging ecosystem, including but not limited to researchers, bioinformaticians, software developers, policy makers and funding agencies. A synergistic approach, combining top-down strategies from organizational and policy perspectives and bottom-up initiatives driven by community-based innovations, is paramount for fostering an environment conducive to solving the problems at hand. A central aspect of our strategy is cultivating a culture that values and understands the importance of BIA, ensuring buy-in from all the various stakeholders (Fig. 4). We emphasize the following six key actions. (1) Enhancing visibility: active participation of bioimage analysts in scientific conferences and forums not only boosts the visibility of BIA but also facilitates collaboration and the exchange of ideas. (2) Building empathy and collaboration: willingness to learn from one another, letting BIA novices learn from experts and developing a common language are key. Engaging in activities such as pair programming (where researchers write code together) or ‘rescue sessions’ for problematic datasets (where novices attempt to analyze very difficult data, discuss computational methods to improve analysis, and brainstorm changes in sample preparation and/or imaging) can bridge the gap between bioimage analysts and researchers. (3) Highlighting unique contributions: by presenting case studies and research outcomes that were made possible exclusively through advanced BIA, we can underscore the unique value it brings to scientific discovery. These success stories can serve as powerful testimonials to the crucial role of quantitative analysis. (4) Advocating for standards in publishing: lobbying for journals to mandate not just the inclusion of quantitative image analysis but also a thorough description of BIA methodologies. Journals should adopt BIA-inclusive publication checklists (Schmied et al., 2024) to ensure that all image quantification is performed according to field standards, similar to existing checklists for statistical analysis. Journals or journal sections dedicated to methods and resources should accept BIA tools and workflows. (5) Securing support from funders: pushing for funding bodies to recognize and support the infrastructure of the BIA community, including the development of communal resources and spaces for collaboration. (6) Emphasizing the importance of user support: advocating for consideration of user support as fundamental to software development and providing funding for tool maintenance ensures that tools are not only technically robust, but also user friendly and suitable for widespread adoption.

Despite these challenges, the BIA community is motivated by a shared sense of organization and purpose and has high enthusiasm and readiness to contribute to the collective mission of enhancing the rigor, reproducibility and impact of scientific research. The recent founding of GloBIAS, a global society for bioimage analysts, is likely to be as globally catalytic in the future as NEUBIAS has been for Europe. The GloBIAS website (www.globias.org) lists training materials, events and pages where biologists can find local BIA experts available for collaboration. Many groups primarily focused on bioimaging [such as BioImaging North America (BINA), the Royal Microscopical Society (RMS), the African Bioimaging Consortium (ABIC), Euro-Bioimaging and Global BioImaging] also now host BIA subgroups and run workshops on BIA training for beginners. The Scientific Community Image Forum (forum.image.sc) also serves as a global community gathering location for image analysis events and education that is free and open to all (Rueden et al., 2019).

Meetings such as The Company of Biologists ‘Effectively Communicating Bioimage Analysis’ Workshop, which inspired this Perspective, play a crucial role in stimulating this community and equipping participants with the knowledge, skills and networks necessary to advocate for and implement best practices in BIA. As we look to the future, it is imperative that we continue to nurture this collaborative spirit by fostering open dialogue, knowledge exchange and innovation. By doing so, we can collectively surmount the challenges that lie ahead, paving the way for groundbreaking discoveries that will propel the field of biomedical research forward. In this spirit of unity and determination, we extend an open invitation to all stakeholders to join us in this endeavor. Together, we are poised to make a substantial impact on the advancement of science, underpinned by the power of effective BIA.

In conclusion, the journey ahead is undeniably challenging yet filled with immense potential. Armed with a clear vision, a robust framework for collaboration and a relentless drive for excellence, the BIA community is well-equipped to embrace the complexities of the future. Let us proceed with confidence and collective resolve, committed to the pursuit of scientific innovation and the advancement of human health.

## Supplementary Material

10.1242/joces.262322_sup1 Supplementary information

Table S1. BIA financial incentive template.
