# Supplementary material for: The crucial role of bioimage analysts in scientific research and publication
Source: J Cell Sci. 2024 Oct 30;137(20):jcs262322. doi: 10.1242/jcs.262322 (PMC11698046; doi:10.1242/jcs.262322)
Supplement: Supplementary information [file joces-137-262322-s1.pdf]

Table S1. BIA financial incentive template.

Available for download at
https://journals.biologists.com/jcs/article-lookup/doi/10.1242/jcs.262322#supplementary-data

Journal of Cell Science • Supplementary information
